# Beyond benchmarks: a narrative review of evolving quality of life frameworks for children with neurodevelopmental and learning disabilities and the need for integrated digital twin approaches

**DOI:** 10.3389/fpubh.2026.1930098

**Published:** 2026-09-30

**Authors:** R Sterling Snead

**Affiliations:** 1 Capitol Technology University, Laurel, MD, United States; 2 Research Department, The Self Research Institute, Broken Arrow, OK, United States

**Keywords:** convention on the rights of persons with disabilities, digital twin, ecological momentary assessment, family quality of life, intellectual disability, learning disabilities, neurodevelopmental disabilities, quality of life

## Abstract

Beginning with an overview of the current state of Quality of Life (QoL), this narrative review provides a synthesis of thirty articles published between 2007 and 2026 to provide insight into the evolution of QoL frameworks, how QoL data tracking has been operationalized by these frameworks, and where the gaps are. While the literature has moved from a focus on deficits and impairment to a strengths-based approach centered around the child, and aligned with the principles of the United Nations Convention on the Rights of Persons with Disabilities (CRPD), one theoretical framework (Schalock & Verdugo’s Eight Domain Model) was identified as being used most often by researchers. In addition, several instruments were also identified as commonly used to measure QoL, which include generic health related QoL frameworks, family QoL frameworks, and Ecological Momentary Assessment (EMA). However, several recurring limitations were identified across the reviewed frameworks, including: (1) use of proxy reports; (2) static or cross-sectional design; (3) fragmentation in terms of number of domains assessed, informant type and assessment methodology; (4) lack of sensitivity to changes in QoL over time in children who experience severe levels of disability; and (5) large gaps between new technology available to support QoL assessments and traditional periodic assessments. Therefore, the reviewed literature suggests that no single framework adequately captures the full range of factors influencing QoL for children with neurodevelopmental and learning disabilities, highlighting the need for a more integrated and comprehensive approach. As such, the findings suggest that digital twin approaches may provide a promising direction for the development of next-generation QoL frameworks. No study identified in this review applied a digital twin approach to QoL assessment in this population. Digital twin approaches are therefore considered a prospective direction for further investigation rather than an empirically established solution, with their potential application to pediatric QoL requiring future implementation and validation.

## 1 Introduction

Quality of Life (QoL) has become an essential concept in disability research, clinical practice, and health policy over the last twenty years (1–3). For children with neurodevelopmental and learning disabilities, including intellectual and developmental disabilities (IDD), autism spectrum disorder (ASD), cerebral palsy (CP), specific learning disabilities (SLDs), and rare genetic disorders, QoL frameworks provide a unifying perspective to go beyond the traditional clinical, functional, and academic indicators (4, 5). In this review, neurodevelopmental and learning disabilities are considered related but not interchangeable categories. The terminology is used to reflect the populations described in the reviewed literature, including conditions affecting neurodevelopment as well as specific difficulties in learning and academic functioning. This represents a larger shift in the disability science paradigm: from a focus on reducing deficits, to using child-centered, strength-based approaches that emphasize the importance of emotional well-being, social participation, self-determination, and the realization of universal human rights for meaningful outcomes (2, 6).

A second way in which QoL is becoming more central is through its application in policy and practical settings. Data collected about QoL (often as part of support need assessments) are increasingly being utilized for developing Individualized Educational Plans (IEPs), providing direction for intervention strategies, determining levels of support services to be allocated to individuals and families, and informing policies that impact hundreds of millions of children and families world-wide (7). However, significant theoretical and methodological challenges remain. Existing frameworks vary in their coverage of disability populations and in the extent to which environmental factors are considered alongside clinical and functional information. In addition, many commonly used QoL assessment approaches rely on cross-sectional measurement and proxy reporting, which may limit their ability to represent the subjective, dynamic, and contextual nature of QoL (8–10).

In this regard, it should be emphasized that the limitations described above reflect the fundamental structure of modern QoL assessment. That structure was developed at a point in time when periodic, paper-and-pencil, proxy-reported assessments were the best options available. Given recent advancements in digital health, ubiquitous computing, ecological momentary assessment (11), and computational modelling, it appears possible to develop a new model for tracking QoL that captures the dynamic, multi-dimensional, and ecologically embedded aspects of children’s experiences in ways that periodic measurement does not. Within this context, a digital twin can be understood as a dynamic, individualized digital representation that is updated over time using data from multiple sources. Unlike approaches focused primarily on data collection or periodic assessment, the digital twin concept emphasizes an evolving representation of the individual that can reflect changes across relevant dimensions over time. To date, however, these capabilities have not been consistently integrated with established QoL theories within a unified framework. Therefore, the purpose of this review is to examine this conceptual-technical gap.

To meet this objective, this narrative review will integrate and compare current knowledge regarding evolving QoL frameworks for children with neurodevelopmental and learning disabilities, including how each operationalizes data collection and tracking. More specifically, this review will address five research questions: (a) How have QoL frameworks for children with neurodevelopmental and learning disabilities evolved, especially concerning the movement from deficit-based to strength-based approaches? (b) What assessment frameworks and instruments have been developed and validated? How do they vary in their methods for collecting data? (c) Are these frameworks consistent with Convention on the Rights of Persons with Disabilities (CRPD) principles? (d) What limitations in data-tracking frameworks currently limit the field’s ability to fully assess and respond to the complex nature of children’s QoL? (e) What conceptual rationale exists for a more comprehensive QoL framework, and how can emerging digital technologies help facilitate this?

Wieringa’s (12) design science research methodology is used as the methodological grounding for this review. According to this methodology, the first step in conducting research involves analyzing existing knowledge to define design issues and problems and determine design requirements for future artifact development. Thus, by identifying the shortcomings of current frameworks and evaluating them collectively, this paper establishes the theoretical foundation for developing an integrated QoL framework(s). As this review concludes without recommending or specifying a particular architecture for an integrated framework, this aspect of that work will be completed in a separate companion article.

## 2 Method

### 2.1 Search strategy and selection

A structured narrative synthesis was conducted using a conceptually guided selection and extraction approach. Literature was identified through targeted searches of PubMed and Scopus databases, supplemented by manual examination of reference lists and selective use of Google Scholar for grey literature and contextual sources. Search terms combined keywords related to QoL (e.g., “quality of life,” “QoL,” “health-related quality of life,” “family quality of life”) with disability and pediatric terms (e.g., “children,” “adolescents,” “neurodevelopmental disabilities,” “intellectual disability,” “autism,” “cerebral palsy,” “Down syndrome,” “learning disabilities,” “rare disease”) and methodological keywords (e.g., “assessment,” “validation,” “psychometric,” “intervention,” “framework”). Boolean operators were used to combine terms with appropriate precision. The focused PubMed and Scopus searches were used to identify contemporary literature, while the broader narrative synthesis also incorporated earlier foundational studies and relevant subsequently identified literature where necessary to trace framework development and address the review questions. The database-specific search strategies, filters, retrieval counts, and source-identification pathway are documented in Supplementary File S5, with the source-identification and selection summary provided in Supplementary File S1. The rationale for retaining individual studies is provided in Supplementary File S2.

### 2.2 Inclusion and exclusion criteria

Studies were included if they met the following criteria: (a) investigated how QoL was conceptualized, measured, or intervened upon in youth with neurodevelopmental/learning disabilities; (b) were published in a peer-reviewed journal or scholarly volume; (c) fell within the broader 2007–2026 publication period considered in the narrative synthesis, including foundational and subsequently identified literature relevant to the development of QoL frameworks; and (d) made important theoretical, empirical, or methodological contributions toward answering the research questions.

The 2007–2026 publication window was selected to capture approximately two decades of QoL framework development while maintaining relevance to the contemporary technological context considered in this review. The period also encompasses broader developments in data technologies, including the increasing use of graph-based approaches for integrating and representing complex data. Within this broader context, Li et al. (13) described the formalization of personal informatics within human–computer interaction research. Relevant foundational literature predating the focused database-search period was retained where necessary to trace the conceptual development of major QoL frameworks.

Therefore, studies that involved adult populations, study conditions outside of pediatric neurodevelopmental/learning disabilities or study outcomes other than those related to QoL were excluded.

### 2.3 Analytical approach

Because the objective of this review was to map the conceptual problem space and identify recurring design requirements rather than to perform quantitative aggregation, a purposive selection strategy was adopted. The focused database searches and manually identified sources were consolidated into a broader source-identification dataset, from which 30 articles were purposively retained for the analytical synthesis as representative of major conceptual contributions, framework innovations, and contextual considerations relevant to the review questions. A three-layer extraction strategy was applied: (a) inclusion rationale mapping, documenting why each study was retained; (b) thematic mapping, identifying recurring conceptual and methodological themes; and (c) gap mapping, identifying limitations relevant to the design of future integrative frameworks. The inclusion rationale for the retained studies is documented in Supplementary File S2, the thematic mapping underlying the synthesis in Supplementary File S3, and the mapping of observed limitations to the derived gap categories in Supplementary File S4.

The synthesis combined conceptual and qualitative interpretation, with thematic categories developed iteratively. Categories included the conceptual evolution from deficit-based to strength-based models, generic and condition-specific measurement frameworks, family QoL frameworks, rights-based perspectives, psychometric properties, cross-cultural validation, technology-supported assessment, and intervention applications. Consistent with Wieringa’s (12) design science methodology, the synthesis was oriented toward identifying problems and design requirements that should inform the development of future frameworks. To facilitate comparative interpretation across the reviewed literature, Table 1 summarizes the major pediatric quality of life (Pediatric QoL) frameworks, their conceptual orientations, methodological characteristics, strengths, and recurring limitations identified through the synthesis.

**Table 1:** Qualitative literature synthesis based on thematic coding and identified design problems.

Literature theme	Evidence from reviewed studies	Observed limitations	Gap type	Design problem identified	Implications for framework design
QoL measurement and instrument validation	Many studies use standardized HRQoL instruments and clinical scales to measure wellbeing in children with special needs.	Measurement approaches vary significantly across studies and often rely on proxy-reported assessments rather than direct child input.	Measurement gap	Lack of a consistent QoL measurement framework across studies.	Need for an integrated QoL measurement structure that can organize multiple measurement dimensions.
Family and environmental context	Research frequently highlights the influence of caregiver support, educational environments, and social inclusion on QoL outcomes.	Contextual factors are often analyzed separately from clinical or functional measures of wellbeing.	Integration gap	Fragmented representation of contextual determinants influencing QoL outcomes.	Framework must incorporate multi-level contextual factors including family, education, and social environment.
Neurodiversity and rights-based perspectives	Some studies critique deficit-based medical models and emphasize acceptance, identity, and environmental accommodation for neurodiverse individuals.	Conceptual discussions are rarely connected to operational measurement models used in empirical studies.	Conceptual gap	Gap between theoretical QoL perspectives and measurable constructs used in research.	Framework should bridge conceptual QoL perspectives with measurable QoL dimensions.
Intervention and technology-based approaches	Emerging research explores assistive technologies, digital health tools, and AI-supported interventions aimed at improving adaptive functioning and daily living skills.	Evidence remains limited and often short-term, with few studies evaluating long-term QoL outcomes.	Implementation gap	Lack of integration between intervention evidence and QoL evaluation frameworks.	The framework should support systematic evaluation of interventions and their impact on QoL outcomes.
Longitudinal and developmental perspectives	A small number of studies examine QoL development across different life stages of childhood and adolescence.	Most research relies on cross-sectional designs that do not capture long-term QoL changes.	Longitudinal gap	Limited understanding of how QoL evolves across developmental stages.	Framework should support longitudinal analysis of QoL development.
Child self-report and proxy reporting	Many studies rely on caregiver or parent proxy reports to evaluate QoL outcomes, while fewer studies capture direct perspectives from children with special needs.	Proxy reporting may not fully reflect children’s subjective experiences, preferences, and priorities.	Measurement gap	Limited representation of the child’s own voice in QoL assessment.	Framework should incorporate mechanisms to capture child-centered QoL perspectives alongside caregiver observations.

This table presents the qualitative synthesis of the reviewed studies based on the controlled thematic coding framework applied during the literature analysis. Each row corresponds to one thematic code used during the review. For each coded theme, the table summarizes the evidence reported in the literature, the limitations identified across studies, the associated gap type, the resulting design problems, and the implications for framework development.

### 2.4 Limitations of the method

This review is subject to limitations arising from its conceptually guided narrative design. The non-systematic and purposive nature of study selection may have introduced selection bias. To provide structure and reduce the potential for arbitrary selection, the review applied predefined eligibility criteria, identified literature across multiple sources, selected studies in relation to the stated research questions, and documented the rationale for inclusion together with the thematic and gap-mapping processes. Nevertheless, these procedures cannot eliminate the potential for selection bias inherent in a purposive narrative synthesis (see Table 2).

**Table 2:** Thematic coding framework and distribution of included studies.

Literature theme	Controlled thematic category	Coding focus	Coding basis (how studies were classified)	Number of studies	Studies included
QoL measurement and instrument validation	Assessment instruments	Assessment instrument validation	Studies focusing on the development, validation, or application of standardized HRQoL instruments for children with neurodevelopmental conditions.	8	Alessi et al. (2025) (38), Schwartz et al. (2025) (39), van Silfhout et al. (2024) (40), López-Espejo et al. (2023) (41), Janssens et al. (2016) (42), Maguire et al. (2023) (43), Mason et al. (2022) (44), and Varni et al. (2007) (45)
Family and environmental context	Family QoL	Family QoL analysis	Studies examining how family support, caregiving conditions, educational environments, and social participation influence the QoL of children with special needs.	7	McCrossin et al. (2025) (46), Miller et al. (2016) (47), Alnahdi et al. (2022) (48), Ten Hoopen et al. (2020) (8), Tarigan et al. (2024) (49), Alenezi et al. (2024) (50), and Ueda et al. (2022) (51)
Neurodiversity and rights-based perspectives	Rights-based alignment	Rights-based alignment	Conceptual and participatory research emphasizing neurodiversity, identity, acceptance, and rights-based interpretations of QoL.	2	Bury et al. (2023) (52) and Leadbitter et al. (2021) (53)
Intervention and technology-based approaches	Policy and implementation	Implementation and policy perspective	Studies investigating assistive technologies, therapeutic interventions, and digital tools designed to improve functional abilities and QoL outcomes.	4	Lamsal et al. (2020) (54), Ten Hoopen et al. (2022) (55), Iannone et al. (2023) (56), and Perry et al. (2024) (57)
Longitudinal and developmental perspectives	Theoretical frameworks	Theoretical model development	Studies exploring how QoL evolves across developmental stages and how longitudinal assessment contributes to understanding long-term outcomes.	7	Alehagen et al. (2025) (58), Bertelli et al. (2020) (59), Epstein et al. (2019) (60), Mahdi et al. (2018) (61), Le et al. (2025) (62), Mahjoob et al. (2024) (63), and Pellicano et al. (2022) (64)
Child self-report and proxy reporting	Measurement challenges	Measurement gap discussion	Research highlights the methodological challenges of relying on proxy reports and the need to incorporate children’s own perspectives in QoL evaluation.	2	Shyu et al. (2025) (65) and McPhee et al. (2023) (66)

This table summarizes the distribution of the 30 retained studies across the thematic codes used during the analytical coding stage of the literature review. Detailed study identification, inclusion rationale mapping, thematic coding, and gap mapping are provided in Supplementary Files S1–S4. Each study was assigned to a thematic code based on its primary research focus, and the resulting coding structure informed the qualitative synthesis presented in Table 1. Additional foundational, contextual, and supporting literature cited throughout the narrative was used to inform interpretation but was not part of this 30-study analytical coding dataset.

No formal study-level quality appraisal or risk-of-bias assessment was undertaken. This methodological choice reflects the purpose of the review, which was to map the conceptual problem space and identify recurring design requirements rather than to quantitatively aggregate outcomes or formally grade the strength of intervention evidence. Consequently, the findings should be interpreted as a conceptual and qualitative synthesis of the selected literature rather than as a systematic assessment of the overall strength or certainty of the evidence. The narrative approach nevertheless enabled detailed thematic examination of the conceptual perspectives, frameworks, and recurring limitations relevant to the objectives of the review.

## 3 The evolution of quality of life frameworks

The 30 articles retained for the analytical coding and narrative synthesis encompassed conceptual and empirical literature addressing the evolution, measurement, and application of QoL in children with neurodevelopmental and learning disabilities. The thematic analysis identified recurring areas concerning the transition from deficit-based to strength-based and person-centred models, multidimensional QoL frameworks, rights-based and contextual perspectives, measurement approaches, family context, technology-supported assessment, and longitudinal considerations. The following sections synthesize these themes, beginning with the conceptual evolution of QoL frameworks and their implications for assessment.

### 3.1 From deficit-based to strength-based models

The evolution toward QoL frameworks for children with neurodevelopmental disorders (NDDs), learning disabilities (LDs) and their families has mirrored the broad trend in disability science. Deficit-focused models of disability dominated early work. These deficit-focused models of disability defined disability solely as a set of problems to be addressed. They evaluated outcomes through measures of reduced symptoms, limitations in function, and development of “normal” skills. Children with NDD/LD were primarily viewed as having deficits compared to the non-disabled population. There was minimal focus on the children’s own experiences, their desires and preferences, and how they could have been better supported.

As of the end of the last century, and significantly throughout the first decade of the twenty-first century, there was a paradigm shift from a deficit model to one focused upon strengths and individual and child-centeredness. Several factors contributed to this shift: (1) Research demonstrated that individuals with disabilities reported similar levels of happiness/well-being regardless of whether they had higher or lower degrees of objective measures of physical and mental impairment; (2) Self-advocacy movements emerged within the disability community; (3) Positive psychology gained prominence in academia and research, including an emphasis on flourishing as much as eliminating pathology; and (4) Social model and biopsychosocial models began to replace medical models in terms of disability-related thinking. QoL is now conceptualized in three ways: (1) Multi-dimensionally; (2) Person-Centered/Individual; and (3) Contextual. Contemporary QoL frameworks are concerned with what goes well in a child’s life and what facilitates his/her flourishing, whereas earlier QoL frameworks were primarily focused upon what does not go well.

This evolution is critical for children with NDD/LD because many times, their developmental paths diverge qualitatively (as opposed to quantitatively) from that of typically developing peers. If we use a deficit model, it may be possible to label legitimate developmental diversity as pathological, and we will fail to recognize the strengths, interests, and capabilities of these children which are important contributors to their meaningful QoL. Conversely, using a strength-based approach enables us to view children as agents who have needs, desires, abilities, and aspirations that can be measured, appreciated and supported within the contexts of available support systems.

### 3.2 The Schalock and Verdugo eight-domain model

The most commonly accepted contemporary QoL framework used in disability research today is Schalock and Verdugo’s (5) eight-domain model. The model suggests that there are eight primary areas where an individual can experience QoL. These include: emotional well-being, interpersonal relationships, material well-being, personal development, physical well-being, self-determination, social inclusion and rights (2, 3). Each area has indicator measures for assessing each area at the individual level, while also being aware of how environment and context affect an individual’s QoL. While these areas are conceptually independent from one another, they are interconnected. This allows researchers to assess specific areas individually or integrate assessments across multiple areas.

There are several foundational assumptions built into this model. One assumption is that QoL is a universal construct, regardless of whether someone has a disability or not. Although people with disabilities will typically assign different weights to various areas based on their unique circumstances, and utilize alternate ways to achieve those areas of QoL, the structural nature of QoL is assumed to exist similarly across all populations (14). A second assumption is that the model supports a person-centered approach. This means that researchers should consider individual preferences, choices, and experiences when using objective measures to measure QoL. The third assumption is that it uses a social-ecological perspective. It recognizes that QoL is influenced equally by individual attributes (i.e., age, cognitive abilities, communication skills) and environmental contexts (i.e., family, school, community, society). Lastly, the model includes rights as a separate domain area rather than treating rights as something outside of QoL, thereby providing explicit support for rights-based principles.

### 3.3 Alignment with the international classification of functioning, disability and health

Contemporary QoL frameworks increasingly integrate elements from the World Health Organization’s (15) International Classification of Functioning, Disability and Health (ICF). The ICF offers a biopsychosocial model that considers health conditions, body structures and functions, activities, participation, and environmental and personal factors, all conceptualized as dynamically interacting components (16). Integration of ICF principles with QoL models enables researchers and practitioners to evaluate not only individual functioning, but also the environmental supports and barriers that shape participation and well-being.

As an illustrative example, a child with cerebral palsy may be assessed in terms of motor body functions, self-care activity limitations, and participation restrictions in school and community settings. The interaction of these dimensions, mediated by assistive technologies, adaptive environments, and family supports, can then be examined in relation to broader QoL domains such as physical well-being, social inclusion, and self-determination. Such integration provides a more comprehensive picture than functional assessment alone and aligns with the goal of person-centered support planning.

### 3.4 Rights-based conceptualization

A major shift recently occurred in QoL theory with the addition of rights-based perspectives based upon the Convention on the Rights of Persons with Disabilities (CRPD) (6), which are discussed at length in several papers (2, 3, 14). The rights-based perspective includes a variety of rights, including privacy, dignity, equality, protection from discrimination, support for advocacy, due process, and the right to participate within one’s community/civic society. Conceptually, this formulation transforms enhancing QoL into a social-justice issue rather than an individualized clinical outcome. Therefore, it shifts the focus of interventions from solely addressing individuals’ deficits towards removing systemic barriers and eliminating discriminatory practices.

The implications of the rights-based perspective have many impacts on both assessing and intervening to enhance the QoL. Specifically, it encourages individuals to make decisions about their own lives instead of having others decide for them (self-directed vs. substitute decision-making); it directs services to dismantle barriers rather than simply treat deficits individually, and it defines enhancing QoL as inherently relational and context-dependent (2). Additionally, the rights-based perspective places a duty on service delivery systems and policy development to create policies that provide rights along with functional outcomes. Further, the rights-based perspective provides additional rationale for applying the strengths-based orientation to QoL assessments because children are treated as rights holders rather than solely as consumers of care.

### 3.5 Neurodiversity and acceptance-oriented perspectives

While much less formalized than Schalock and Verdugo’s (5) model of QoL, models based on a neurodiversity orientation are beginning to impact how QoL is conceptualized, especially regarding autistic people (17, 18). Models based on a neurodiversity orientation dispute medical model assumptions that define disability solely as a problem to be fixed, rather than defining it in terms of identity, acceptance, or environment, which can contribute to an individual’s well-being. For example, McConachie et al. (17), found that the process of developing QoL assessments by including autistic people was important because it allowed the dimensions of QoL measured by those assessments to be related to the dimensions of QoL that autistic people perceived were relevant.

Overall, the theories discussed above represent a comprehensive approach to QoL that includes a focus on the positive aspects of a person’s life, is centered on each individual, views the world from multiple ecological levels, and promotes social justice. The theories also represent a conceptual base for many of the measurement tools developed since then. However, as described below, the translation of this conceptual sophistication into measurement approaches capable of integrating multidimensional, contextual, and longitudinal QoL information remains limited.

## 4 Frameworks for tracking quality of life data

Developing quantitative methods from QoL theory means creating practical methods to select assessments, collect data, score it, and interpret results. This review illustrates at least five overlapping families of methods identified across the reviewed literature that researchers and practitioners are currently using to assess QoL: (1) Generic Health-Related Quality of Life (HRQoL) Methods, (2) Disability Specific Generic Methods, (3) Condition Specific Methods, (4) Family Quality of Life (FQoL) Methods, and (5) Technology-Supported Assessment Methods. Each method will address different measurement priorities than the others, as each will include unique strengths and weaknesses.

### 4.1 Generic health-related quality of life instruments

These are instruments that measure generic aspects of children’s HRQoL. Examples include the KIDSCREEN family (the KIDSCREEN-10, the KIDSCREEN-52, the KIDSCREEN-27), the Pediatric Quality of Life Inventory (PedsQL), and the Child Health Questionnaire (CHQ). These instruments can be applied across both general and disability-related populations, but can be used for comparisons across health conditions and when compared to norms from the general population (19, 20). For instance, the KIDSCREEN-27 is able to measure dimensions of children’s physical, psychological, and social well-being broadly. It was shown to be validly translated and culturally adapted for use across many European countries (20). Another example of an important generic instrument is the Patient Reported Outcome Measurement Information System (PROMIS) which provides high-quality item banks in multiple areas of health and computerized-adaptive testing capabilities (21).

The benefits of generic methods are that they allow for comparison among health conditions and with their non-disabled peers; they facilitate population-based studies; they have larger normative samples; and they have undergone rigorous psychometric development. In addition, they provide a common vocabulary regarding QoL across various clinical settings.

However, generic methods have limitations in capturing aspects specifically related to disability experience and support. Some dimensions relevant to children with neurodevelopmental and learning disabilities may therefore be less comprehensively represented by these measures (17). Generic instruments may not fully capture disability-specific experiences, particularly among children with significant cognitive, communication, or motor impairments. Accordingly, generic measures may need to be complemented by disability- or condition-specific approaches when a more comprehensive assessment is required.

### 4.2 Disability-specific generic frameworks

Generic frameworks that are specific to disability are aimed at addressing QoL across a variety of different types of disabilities, but can be more limited in their application. However, they provide a balanced approach between breadth of use and relevant aspects to individuals experiencing various forms of disability. There are two frameworks that are primarily utilized in this area: the KidsLife Scale and the Quality of Life Inventory — Disability (QI-Disability).

#### 4.2.1 KidsLife scale

The KidsLife Scale is a widely validated QoL instrument for children and adolescents with IDD. This framework is grounded in the Schalock and Verdugo eight-domain model and provides both an age- and ability-based version of the tool to account for both development and function (3, 22). Gómez et al. (22) completed a large sample size psychometric study utilizing the KidsLife Scale in evaluating children and youth with Intellectual Disability (ID) in Spain. A confirmatory factor analysis supported the eight-domain structure, and internal consistency ranged from 0.812 to 0.949 across all domains. The items in the KidsLife Scale were rated by proxy respondents (i.e., typically parents, teachers or caregivers) using behavioral frequency anchors (never, sometimes, frequently, always) which focused on behaviors and events that can be observed as opposed to subjective states inferred by the respondent. As examples, items in the emotional well-being domain assessed whether the child exhibited pleasure, satisfaction and had positive affect and had opportunities for preferred activities as opposed to assessing how happy the child felt internally (22). Additionally, normative data provided by validation studies allow for comparison of an individual’s QoL profile to those of peers, who are also referenced to either age or type of disability as needed for more effective intervention planning (2, 3). Therefore, the KidsLife Scale is an established tool in pediatric IDD QoL assessment and has been adapted across multiple cultural and linguistic contexts.

#### 4.2.2 KidsLife-down syndrome scale

Given that generic IDD instruments do not adequately capture the experiences or support needs of children with Down syndrome, Gómez et al. (14) adapted the KidsLife Scale to create the KidsLife-Down Scale. They then performed a validation study on 405 children with Down syndrome in Spain. McDonald’s Omega was found to be acceptable across domains (0.85 to 0.90), and the intercorrelated-eight-domain model produced the best fit to the data. Thus, Gómez et al. (14) demonstrated the benefits of combining a common theoretical framework with population-specific item modification to increase sensitivity to measurement within unique disability groups, yet maintain a coherent conceptual relationship with the larger body of QoL literature (1, 14).

#### 4.2.3 Quality of life inventory—disability (QI-disability)

The QI-Disability is a validated generic framework developed in Australia that has been implemented with children with Rett syndrome, ASD, CP, and rare epilepsy syndromes such as Lennox–Gastaut and Dravet syndromes (4, 23). Downs et al. (4) investigated the psychometric characteristics of the QI-Disability in 253 children with diverse IDD using both Classical Test Theory (CTT) and Item Response Theory (IRT) frameworks. The QI-Disability showed strong performance across all six domains of QoL: positive emotion, negative emotion, social interaction, leisure activity, independence, and family interaction.

One of the major strengths of the QI-Disability is its ability to assess a broad range of levels of functioning, including those with severe/profound levels of impairment who are often excluded from QoL research due to difficulty measuring their level of functioning (22). The QI-Disability utilizes proxy-reporting methods focusing on observable behaviors such as participation in activities, expressed emotions, etc., and measures these behaviors as evidenced by McGrath et al. (24) who demonstrated that the QI-Disability could measure clinically significant changes in QoL in children with complex needs after receiving specialist play interventions, thus providing evidence for its utility as an outcome measure in intervention research. Further validation studies have extended the application of the QI-Disability to children with Lennox–Gastaut Syndrome and Dravet Syndrome, demonstrating sensitivity to seizure frequency, seizure severity, and side effects resulting from medications taken to control seizures (23).

### 4.3 Condition-specific frameworks

Condition-specific frameworks are designed to examine QoL at the level of a specific condition (e.g., cerebral palsy), allowing greater depth and sensitivity to population-specific experiences and support needs while limiting cross-condition comparisons.

#### 4.3.1 Cerebral palsy quality of life questionnaire

The Cerebral Palsy Quality of Life Questionnaire (CP QoL) is used to assess the QoL of children and adolescents with cerebral palsy. It includes questions related to pain, activity limitation, participation and social well being (9, 25). According to Badia et al. (25), who adapted and validated a Spanish version of the CP QoL, it had strong psychometric properties, with internal reliability ranging from 0.74 to 0.93 across the various domains of the tool. Hutson et al. (9) reviewed QoL outcome measures for children with severe cerebral palsy receiving postural care and identified limitations in the sensitivity of available measures for detecting clinically meaningful changes in this population. The authors therefore highlighted the need for QoL measures capable of detecting small but clinically relevant changes in function.

#### 4.3.2 Autism-specific quality of life frameworks

Numerous QoL frameworks have been developed specifically for individuals with ASD. Many focus on aspects of QoL experienced uniquely by individuals with ASD such as sensory issues, social communication difficulties, restrictive/repetitive behaviors and anxiety (8, 17, 18). McConachie et al. (17) worked collaboratively with autistic adults, family members, and professionals to create an autism-specific QoL measure based on participant input regarding what matters most when assessing QoL. In addition to comparing health-related QoL and care-related QoL in children with ASD and their caregivers, Ten Hoopen et al. (8) found that generic health-related QoL measures may not fully capture the impact of ASD on children and families. Ten Hoopen et al. also stated that both types of QoL should be assessed together because both are vital components of overall QoL.

Developing valid assessments of QoL in minimally verbal autistic children with intellectual disabilities is complicated by several factors. First, there are very few applicable items in traditional QoL measures. Second, researchers continue to debate how accurately one can infer subjective experiences from observable behavior.

#### 4.3.3 Frameworks for profound intellectual and multiple disabilities

Professionals assessing QoL in children with profound intellectual and multiple disabilities (PIMD) face additional barriers compared to other populations due to these children’s limited cognitive abilities, difficulty communicating verbally, and medically fragile nature (10). While some QoL frameworks rely on the child’s ability to communicate through language or exhibit measurable behaviors, most children with PIMD cannot meet these requirements. Therefore, many specialized methodologies have been developed to assess the QoL of children with PIMD, primarily through observational techniques, such as observing children’s alertness, engagement, comfort, and display of positive affect over time. However, even these specialized methods present problems. As Nieuwenhuijse et al. (10) identified in their narrative review of QoL assessment strategies in children with PIMD, a major concern regarding using these observational techniques is that different observers will report different levels of each variable. Thus, Nieuwenhuijse et al. (10) recommended that multiple observers and data collection methods be used to increase confidence in the validity of subjective reports in this group.

#### 4.3.4 Frameworks for rare genetic and neurological conditions

Researchers have recently begun developing new QoL frameworks for children experiencing rare genetic and neurological disorders including Dravet Syndrome, Lennox Gastaut Syndrome, and Rett Syndrome (4, 23). Each population faces extreme challenges to their QoL as a result of severe impairments in intellectual functioning, high rates of seizures, motor dysfunction and progressive decline in functioning (4, 26). For example, Shah et al. (23) evaluated if measures of QoL could help identify children whose seizures were having a negative effect on their QoL by showing that QI-Disability scores were negatively correlated with seizure frequency and severity, as well as with side effects of medications used to treat seizures. Researchers have noted that because Rett Syndrome involves progressive losses in communication skills, hand movement skills, mobility, and independence, along with nearly universal presence of epilepsy and high incidence of scoliosis and gastrointestinal dysfunction (4), that any framework intended to assess QoL for individuals diagnosed with Rett Syndrome must include consideration for these progressive losses.

#### 4.3.5 Frameworks for children receiving palliative care

While still underrepresented in terms of research, there is growing interest in developing QoL frameworks for children who require palliative care (27, 28). Toro-Perez et al. (27) performed a cross-sectional study examining whether there were any differences between the self-assessment of QoL by children requiring palliative care and their parent’s assessments. Toro-Perez et al. reported finding differences between the two ratings, which reinforced previous recommendations that whenever possible, children’s own ratings should be used when evaluating their QoL. Nagorna et al. (28) pointed out that hand function represents another critical aspect of QoL in non-cancerous pediatric palliative care. Preserving function and promoting comfort are central goals of treatment for patients in non-oncologic pediatric palliative care settings.

### 4.4 Family quality of life (FQoL) frameworks

As research has progressed in evaluating QoL for children with neurological and learning disabilities, it has become increasingly recognized that QoL for children with disabilities cannot be evaluated independently of QoL for their families (FQoL). The empirical definition of FQoL was first established by Brown et al. (29); however, the application of this concept to IDD populations was further explored by Bertelli et al. (30). FQoL pertains to whether family needs are being fulfilled, whether families enjoy spending time together, and whether families are able to achieve their goals together. Additionally, FQoL frameworks provide an opportunity to evaluate the family-level experiences that contribute to child outcomes and vice versa.

Child-QoL and FQoL exist within a reciprocal and dynamic relationship. Factors associated with the child, including characteristics of his/her disability, level of need for support, and behaviors, all impact how a family functions and the amount of stress experienced by parents, ultimately influencing the overall FQoL. Conversely, FQoL factors such as the cohesiveness of the family unit, availability of financial resources, social support systems, and the mental health status of parents, all influence child outcomes via high-quality care giving practices, accessibility to services/supports, and/or the emotional climate experienced by the child (20, 30). This interdependence supports the use of integrated measures of child and family QoL. However, as discussed in later sections, achieving this type of integration remains challenging across existing framework families.

Brown et al. (29) completed a multicounty study examining FQoL among families raising children with disabilities. Results indicated both similar types of challenges faced by families and variations in family experiences associated with culture-specific values, service delivery options, and societal attitudes toward disability. Therefore, FQoL should not be viewed as a universal construct but should be considered within the context of the specific cultural environment in which it exists. Ali et al. (31) used the World Health Organization Quality of Life-BREF (WHOQOL-BREF) (32) to assess parental QoL during the COVID-19 pandemic. Parents of children with disabilities reported lower QoL scores across the four domains of physical health, psychological health, social relationships, and environment, with statistically significant differences observed in physical health, social relationships, and environment. The pandemic exacerbated previously identified vulnerabilities related to disrupted services, increased social isolation, and heightened caregiving burdens. In addition to emphasizing the vulnerability of families to external systemic stressors, these results emphasize the necessity for understanding broader environmental and policy contexts in developing FQoL frameworks.

### 4.5 Technology-supported and ecological assessment frameworks

In addition to traditional periodic instruments used to assess QoL, technology-supported and ecological approaches provide opportunities for collecting information closer to individuals’ everyday experiences. Ecological momentary assessment (EMA), for example, enables repeated assessment of experiences and behaviors in real-world settings and can reduce reliance on retrospective reporting (11). EMA can be used to collect information at multiple time points on aspects of daily functioning, including emotional states, activities, and contextual experiences (11). For children with sufficient cognitive and communication abilities, these approaches may include self-report, whereas assessment of children with profound intellectual and multiple disabilities may require greater reliance on observational or proxy-based approaches (10).

Technology can also support more flexible administration of established assessment instruments. Computerized adaptive testing approaches, such as those developed within the PROMIS framework, use item banks and adaptive item selection to support precise measurement while reducing respondent burden (21). This flexibility may facilitate repeated assessment while adapting the measurement process to the individual respondent. Together, these approaches illustrate how technology-supported methods may complement traditional periodic QoL assessment by enabling more frequent, context-sensitive, or individually adapted assessment.

Technology-supported interventions, including non-invasive neurostimulation approaches such as external trigeminal nerve stimulation, have also been investigated in children and adolescents with neurodevelopmental conditions (33).

## 5 Comparative analysis of existing frameworks

### 5.1 Strengths across frameworks

Collectively, the existing frameworks described here demonstrate a number of important strengths. First, several are theoretically grounded; for example, frameworks based on the Schalock and Verdugo (5) eight-domain model share a conceptually consistent foundation (1, 2). Second, many have undergone psychometric evaluation, with evidence regarding reliability and validity available for the populations for which the measures were designed (3, 4, 14, 22). Third, several have been culturally and linguistically adapted, supporting cross-national comparative analyses (20, 25, 29). Fourth, the reviewed literature includes generic, disability-specific, and condition-specific frameworks, allowing both broad comparisons and targeted evaluations. Fifth, some frameworks have incorporated family-level and rights-based considerations, thereby extending beyond a sole focus on an individual’s level of functioning.

### 5.2 Weaknesses across framework families

Although there are numerous benefits to having multiple QoL frameworks available for use in research studies and clinical applications, a common set of limitations apply broadly across families/frameworks limiting the ability of the field to develop fully integrated QoL frameworks for assessing QoL longitudinally.

#### 5.2.1 Reliance on proxy reports

Many studies on pediatric QoL rely on parent/proxy reports. While proxy reports are often necessary due to a variety of circumstances (e.g., children with severe developmental delays), they tend not to correspond well with child self-reporting when both are available. Parents frequently report lower ratings on QoL than children themselves report (8, 17, 27). It is difficult to determine if discrepancies result from caregiver worry/concern about their child versus their child’s true feelings/experiences. Approaches that improve the correspondence between proxy reports and child self-reports include: (1) promoting greater use of self-report via accessible modes of communication; (2) utilizing multiple proxy reporters/informants across different contexts; (3) teaching proxies to focus on observable behavior (vs. hypothesized internal states); and (4) involving people with disabilities in the process of developing instruments.

#### 5.2.2 Limited cross-sectional designs

While some QoL frameworks include longitudinal designs [e.g., (4)], many QoL frameworks are cross-sectional in design, resulting in “snapshots” that may not fully capture changes over time or the dynamic nature of child well-being (1). Many frameworks limit test-re-test intervals to weeks/months, thereby having limited capacity to account for phenomena that occur rapidly within days or weeks, which may be important for understanding a child’s everyday life (1). Implicitly most models depict static QoL states interrupted by episodic measurements rather than depicting continuous trajectories influenced by dynamic interactions among biological, behavioral, social, and environmental factors.

#### 5.2.3 Overlapping domain and population assessments

The proliferation of domain- and population-specific QoL frameworks has resulted in fragmentation in assessment strategies. A child with cerebral palsy who also has an intellectual disability and seizures may receive assessments across a continuum of generic HRQoL measures, disability-specific measures, condition-specific measures, and Family Quality of Life (FQoL) measures, each producing distinct conceptual structures and prioritizations approaches. Clinicians and planners subsequently aggregate these disparate sources of information, often informally, creating a risk for inconsistencies in assessment and decision-making processes.

### 5.3 Emerging directions for integrated and adaptive QoL frameworks

Emerging trends in QoL research increasingly emphasize adaptability, cross-cultural applicability, family-centered perspectives, and technology-supported longitudinal assessment approaches. These developments attempt to address many of the conceptual and methodological limitations observed across traditional QoL framework families.

#### 5.3.1 Cross-cultural validation and adaptation

There are multiple reasons why cross-cultural validation of QoL scales and other measures used to assess the QoL for individuals with neurodevelopmental and learning disabilities is important. These include the fact that QoL frameworks have been translated and adapted internationally. The KidsLife Scale and QI-Disability have both been adapted for use in different countries and cultures. Cross-cultural validation is needed beyond just translation of the language of the scale. It involves assessing whether concepts and themes associated with QoL exist in similar ways across cultures. Brown et al. (29) and Ravens-Sieberer et al. (20) describe the importance of ensuring that the constructs and domains included in a QoL framework are equally valid across culturally diverse populations. As an example, certain items may require modifications so that they are culturally relevant. In addition, researchers should examine assumptions regarding the degree of autonomy experienced by the population being studied, family roles, levels of community participation, etc. in the culture targeted by the study (29).

#### 5.3.2 Adaptive and rights-based QoL assessment

A number of authors indicate that QoL frameworks can provide a means to evaluate the effectiveness of interventions if the frameworks are capable of detecting true change, yet remain relatively stable when there has been little to no change over time. The QI-Disability has demonstrated this capability of detecting true change related to intervention with significant improvement found in several domains (e.g., positive emotions) post-intervention (24). While this demonstrates that at least one existing QoL framework possesses the required properties to effectively assess the impact of interventions for children with neurodevelopmental and learning disabilities, Hutson et al. (9) state that existing QoL measures may have limited sensitivity for children who have severe disabilities. They argue that small degrees of functional change often result in large increases in an individual’s comfort and overall sense of well-being. Therefore, Hutson et al. further reinforce the call for developing more precise, frequent, and adaptive measurement systems.

As discussed previously, the United Nations established a global human rights treaty for persons with disabilities, known as the CRPD, in 2006. The CRPD defines “disability” as resulting from interactions between an individual’s impairment(s) and barriers in society and attitudes. Further, the CRPD obligates States to guarantee equal access to all rights guaranteed under international law. Many provisions in the CRPD relate to QoL for children with neurodevelopmental and/or learning disabilities. Specifically:

1. Article 7 emphasizes that children have the right to special protection and care, and that their best interests should guide all actions concerning them.
2. Article 12 acknowledges the legal capacity of children with disabilities, stating that children have the right to make decisions independently or with assistance.
3. Article 19 provides for children’s freedom to live in a setting that fosters independence and full community participation.
4. Article 24 asserts that children with disabilities have the right to receive education in an inclusive environment that is accessible to all children.
5. Article 25 recognizes children’s rights to physical and mental health services.
6. Article 30 provides for children’s rights to participate freely in recreational activities and sports.

Schalock and Verdugo (5) developed a model of QoL that includes eight domains: emotional well-being, interpersonal relationships, material well-being, personal development, physical well-being, self-determination, social inclusion, and rights. Their model was originally developed for adults, but has since been adapted to be applied to children with ID/DD (3, 14). Research conducted utilizing Schalock and Verdugo’s model and other frameworks that similarly emphasize rights as a central aspect of QoL for people with IDD suggest that rights-related issues are areas of vulnerability for people with IDD. Gómez et al. (2) report that children with IDD frequently do not consistently experience privacy, autonomy in decision-making and opportunities for meaningful input into life decisions. Such results highlight the need for systematic and policy-based initiatives that transform rights-oriented ideals into actual lived experiences for children with IDD.

The International Disability Alliance defines self-determination as “the ability to take charge of your own life”. Self-determination is a core component of QoL and a key right articulated in Article 12 of the UN Convention on the Rights of People with Disabilities. The practice of supporting decision making (SDM) represents a new approach to facilitating decision making among people with disabilities. SDM seeks to support decision making by providing support to people with disabilities without undermining their legal capacity. SDM presents challenges for families whose members have significant communication limitations or cognitive difficulties. Families must develop strategies for adapting decision-making processes to meet the developmental stage of each member of the family unit. QoL assessments will increasingly contribute to developing self-determination plans tailored to each individual by helping identify an individual’s preference, capabilities and needs for support.

In order to comply with principles outlined in rights-based policies such as the UN Convention on the Rights of Children and the UN Convention on the Rights of People with Disabilities, data collection and management frameworks must prioritize protecting an individual’s dignity, promote his/her agency, and provide mechanisms to safeguard her/his privacy. The child’s perspective must be promoted, taking into account their abilities to communicate and understand information, as well as mechanisms to facilitate them retaining control over data that is collected about them. Similarly, data management systems should ensure equitable access to data resources across different socio-economic groups, geographic locations and cultural backgrounds. Existing QoL frameworks may incorporate some of these principles via ethical research methodologies, but rights-compatible approaches to data collection are not consistently embedded within their design.

#### 5.3.3 Digital twin paradigm for pediatric QoL

Based on the analysis above, the reviewed literature supports the need for a more integrated approach to pediatric QoL assessment. No single framework reviewed here appears to capture the full range of dimensions and temporal, contextual, and person-centred considerations identified across the literature. The recurring themes, limitations, and system-level gaps identified through the comparative analysis therefore point to a set of design requirements for greater integration, as summarized in Figure 1. These findings do not themselves demonstrate that a digital twin is an empirically validated solution. Rather, the digital twin paradigm is considered here as a prospective approach whose characteristics may correspond to several of the requirements identified through the review.

**Figure 1:**
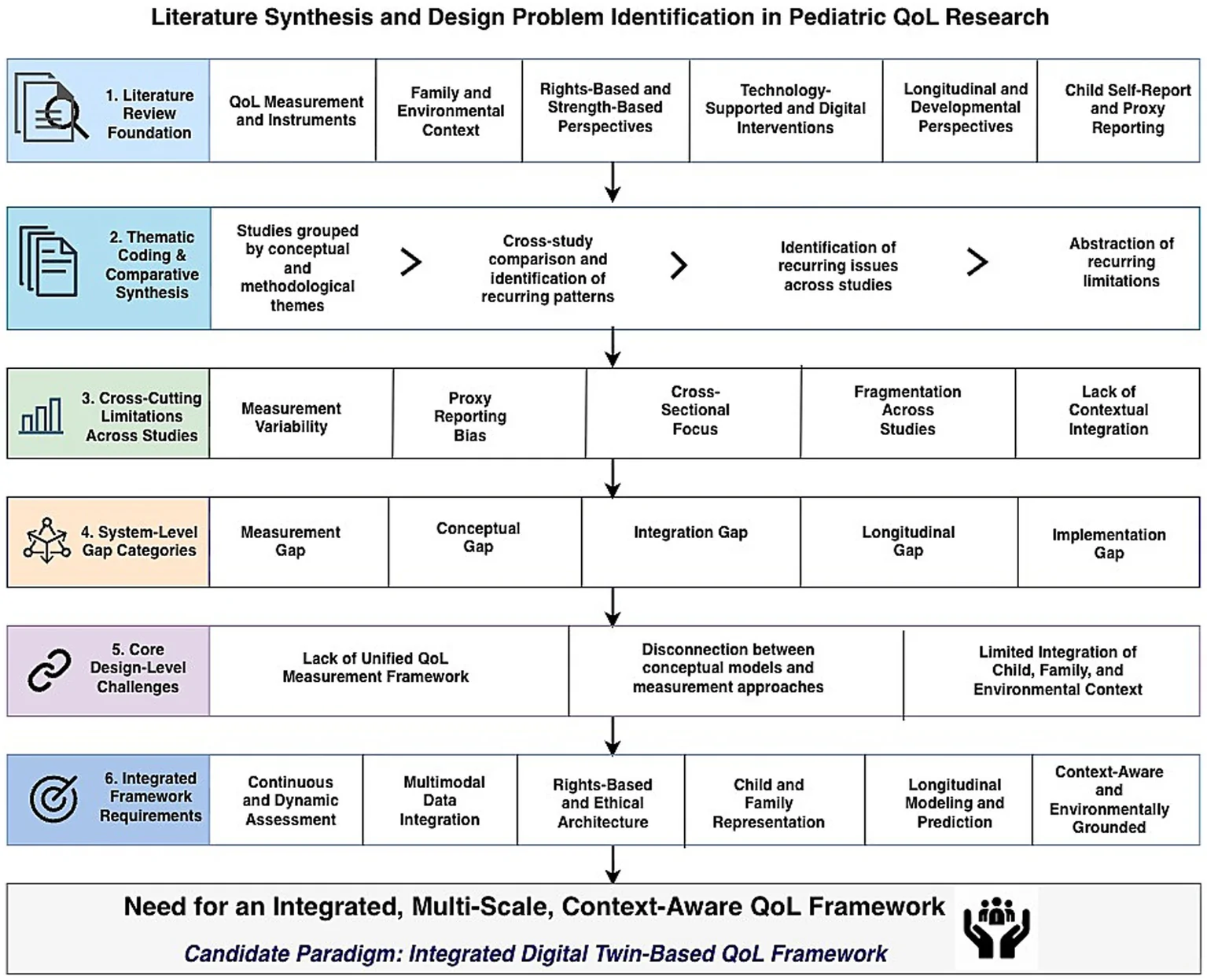
From literature review to design problem identification in pediatric quality of life (QoL) research. The reviewed literature was thematically coded and comparatively synthesized across framework families, resulting in the identification of recurring methodological and conceptual limitations. These limitations were abstracted into system-level gap categories and translated into core design-level challenges. The synthesis highlights the need for an integrated, context-aware, longitudinal, and rights-based QoL framework, for which a digital twin paradigm is proposed as a candidate conceptual approach.

QoL theory currently defines a QoL construct as multifaceted (including biological, psychological, social, and environmental aspects), dynamic (i.e., changing over time), context dependent (i.e., influenced by environmental factors), person-centered (i.e., focused on individual perspectives), and rights-oriented (i.e., emphasizing respect for individual rights). As stated previously, existing QoL frameworks generally attempt to operationalize this complex construct using static, proxy-reported surveys measuring only a limited portion of a child’s QoL within discrete conceptual domains. The disconnect between a theoretical conceptualization of QoL and its operationalization therefore impacts both the validity and the generalizability of QoL assessments that are made for purposes of clinical intervention planning and educational policy formulation.

A number of additional limitations arise from this structural disconnect. One major limitation is that current frameworks may be limited in capturing intra-individual variation in QoL across time and context. Consequently, current frameworks may have limited capacity to support interventions that are responsive to the specific conditions under which a child is likely to thrive or struggle. A second limitation is that current frameworks generally provide limited integration of data from biological, behavioral, social and environmental realms of functioning despite conceptualizations suggesting an interdependent relationship among these realms. A third limitation is that current frameworks may have limited capacity to accommodate emerging technologies for collecting high frequency, low burden ecological data streams. A fourth limitation is that current frameworks may have limited capacity to accommodate children whose communication style does not conform to traditional self-report paradigms. A fifth limitation is that current frameworks do not consistently integrate family-level analyses as an integral rather than secondary component of assessment. A sixth limitation is that current frameworks may have limited sensitivity to rapid developmental change during childhood and adolescence. These six limitations reflect design-level observations derived from the reviewed literature rather than findings from head-to-head comparative testing of QoL frameworks. No study identified in this review directly compared the frameworks across these dimensions; therefore, these observations should not be interpreted as empirical findings about their relative performance.

##### 5.3.3.1 Conceptual foundations of digital twins in healthcare

Digital twin approaches originated in engineering and have increasingly been explored in healthcare as dynamic computational representations that can be updated using multiple data sources. In healthcare contexts, such representations may incorporate biological, behavioral, clinical, social, and environmental information to characterize changes in an individual’s state over time (34). In the context of this review, these characteristics provide a conceptual basis for considering whether digital twin approaches could support more dynamic and integrated representations of pediatric QoL.

These characteristics distinguish the digital twin concept from a system that primarily aggregates or integrates multiple sources of data. In the conceptual use considered here, multimodal data integration provides the inputs to an individualized representation of the child that can be updated as new information becomes available and used to represent changes in health, behavior, and context over time. More advanced digital twin implementations may additionally support simulation or prediction based on the evolving individual model. These capabilities are proposed here as directions for pediatric QoL research rather than as established outcomes of digital twin use in this population.

##### 5.3.3.2 Potential contributions of the digital twin paradigm to pediatric QoL

As previously discussed, several characteristics of the digital twin paradigm may offer potential ways of addressing limitations identified in pediatric QoL assessment. Ecological momentary assessment can support repeated assessment of experiences and behaviors in everyday contexts (11), while other data sources, such as wearable devices, environmental measures, and existing records, could potentially provide complementary information within a future integrated approach. Digital twin approaches may provide a means of integrating such multimodal data into individualized computational representations incorporating biological, behavioral, social, and environmental dimensions (34). In the context of pediatric QoL, these capabilities could potentially support more individualized assessment, consideration of child and family contexts, and longitudinal representation of developmental change. These applications remain conceptual possibilities and have not been established for pediatric QoL assessment.

It is essential to note that a digital twin framework for QoL is not intended to replace existing validated frameworks for measuring QoL. Instead, valid and reliable measures such as the KidsLife Scale and QI-Disability could serve as conceptual anchors and structured measurement points. However, continuous multimodal data could complement these measures by capturing QoL related behaviors between formal assessments. Computerized adaptive testing strategies leveraging PROMIS and other item banks may help reduce respondent burden while supporting precise measurement (21). Thus, a hybrid approach combining elements of both the cumulative body of knowledge surrounding QoL and advanced digital capabilities provides a basis for future investigation.

Applications of digital twin approaches are emerging across healthcare domains, including diabetes and cardiovascular care, where approaches incorporating longitudinal patient data, physiological monitoring, and computational modelling have been explored to support individualized representations of patient state (35). These applications illustrate how dynamically updated, individualized models are being explored in related healthcare settings; however, their application to pediatric QoL has not been established and remains a direction for future investigation.

To illustrate how these characteristics could be considered in a pediatric context, Figure 2 uses asthma as a conceptual example of a person-centred digital twin approach. The example illustrates how medical and health information, including symptoms, digital-inhaler data, physiological measures, clinical records, and wearable data, could be considered alongside environmental information such as indoor air quality, including particulate matter (36), as well as outdoor air quality, weather conditions, location, and other contextual information. Digital inhaler technologies can provide objective information on inhaler use and inhalation characteristics, including the timing and quality of inhalations (37). Together, these information sources could contribute to a continuously updated individualized representation of the child over time, with potential applications in personalized monitoring, identification of environmental triggers, risk assessment, shared decision-making, and longitudinal QoL assessment. Figure 2 is intended as a conceptual illustration rather than a proposed or validated technical architecture. The potential contributions described in this subsection should be interpreted as prospective rather than demonstrated capabilities. Although digital twin approaches may offer features relevant to the limitations identified in current QoL frameworks, their application to pediatric QoL has not been established and remains a direction for future investigation.

**Figure 2:**
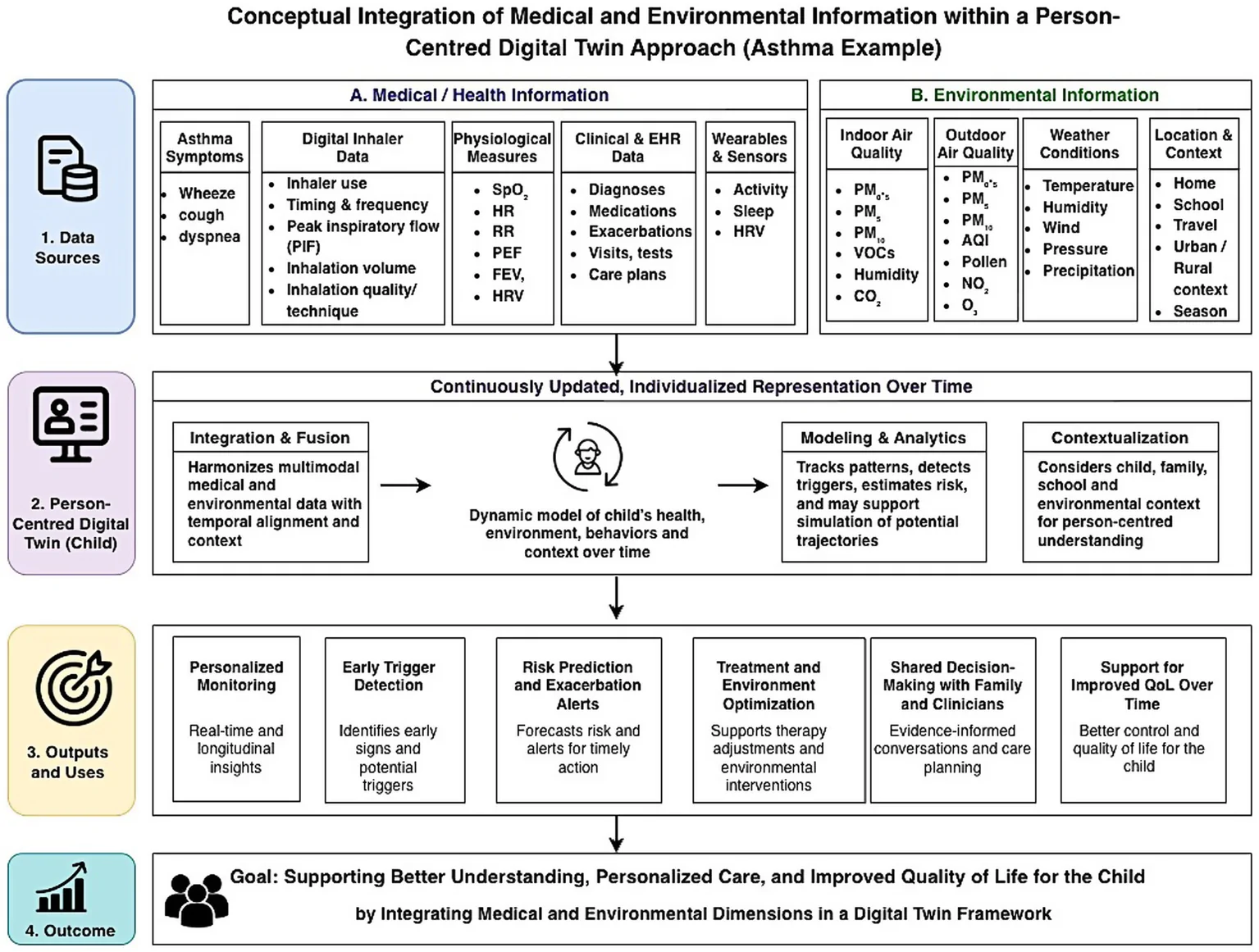
Conceptual integration of medical and environmental information within a person-centred digital twin approach: an asthma example. The figure illustrates how multimodal medical, physiological, clinical, wearable, environmental, and contextual information could contribute to a continuously updated individualized representation of a child over time. Potential outputs are presented conceptually and do not represent an implemented or validated digital twin architecture for pediatric QoL. This figure provides a conceptual illustration, using asthma as an example, of how medical and environmental information could be integrated within a person-centred Digital Twin approach to support longitudinal pediatric quality-of-life assessment.

##### 5.3.3.3 Embedding digital twin frameworks in alignment with strength-based and rights-based principles

For digital twin frameworks for QoL to be aligned with strength-based and rights-based principles in addition to being technologically feasible, strength-based and rights-based principles must become integral components of design decisions. The current strength-based orientation of QoL emphasizes highlighting individual capacities, preferences, and engagement relevant to QoL rather than focusing solely on deficits (1, 3). Rights-based principles emphasize protecting children’s autonomy and rights through frameworks that promote equity, accessibility, and user-centered design and privacy protection (6). Design decisions at all levels, including data modalities, algorithmic decision-making, support tools, enabling technologies, and user interfaces must incorporate these commitments.

Embedding rights-based and strength-based principles in design decisions is not trivial. Ensuring that data collection methods minimize the loss of perspective among minimally verbal children, that proxy mediation does not supplant the child’s voice, and that algorithmic biases do not perpetuate inequality all require deliberate consideration. Operationalizing Supported Decision Making through accessible interfaces and processes that facilitate rather than impede individual agency must be achieved within digital frameworks. Ultimately, the framework should support self-directed growth and flourishing rather than functioning as an instrument of surveillance or control.

##### 5.3.3.4 Methodological, ethical, governance, and feasibility challenges

Developing digital twin frameworks for QoL presents numerous methodological and ethical challenges that would need to be addressed prior to widespread implementation. From a methodological standpoint, establishing whether integrated and computationally derived estimates of QoL correlate well with the lived experience of children, particularly those unable to report on their own experiences will necessitate continued methodological work including cross-validation and triangulation across multiple data sources, methods, and previously established outcome measures (4, 9, 10). Psychometric evaluations (e.g., reliability, validity, sensitivity to change) would need to be adapted to accommodate multimodal and continuously updating computational representations requiring appropriate methodological approaches.

From an ethical standpoint, there exist numerous privacy, consent, and stewardship concerns associated with continuously collecting multimodal data on children with disabilities. Children’s increasing capability for independent decision making must be respected and mechanisms for supported decision making must be built into data architectures (3). Longitudinal monitoring may also raise concerns regarding surveillance and the potential for persistent records to have unintended consequences as children move across healthcare, educational, and social contexts. Data ownership, access, purpose limitation, retention, and stewardship would also require transparent governance arrangements. Additionally, implementing digital twin frameworks for QoL will require resolving issues around data interoperability across healthcare, education, and socioeconomic services; developing ontology standards across different professional fields and cultural contexts (20, 29); ensuring equitable access to technological infrastructure; providing extensive education and training for clinicians, educators, and families to utilize digital twin frameworks appropriately; and mitigating risks associated with over-reliance on quantitative data at the exclusion of qualitative, narrative, and relational understandings.

Beyond these ethical and governance concerns, feasibility will depend on the practical capacity to integrate heterogeneous data sources, maintain sufficiently complete and reliable longitudinal data, support interoperability across settings, and sustain the technical infrastructure required for continuously updated individualized models. Implementation may also impose additional demands on families, clinicians, educators, and service systems. Continuous or passive data collection should not be assumed to reduce burden automatically, as it may introduce additional requirements for device management, data oversight, and consent administration. These considerations mean that the potential advantages of a digital twin approach must be evaluated alongside its technical complexity, resource requirements, acceptability, and accessibility before its value for pediatric QoL assessment can be established.

### 5.4 From conceptual case to concrete framework

The purpose of this article was to establish the case for a comprehensive and complex data framework based on the limitations of current approaches. This paper does not propose a specific architectural model for a new framework. The forthcoming companion paper will articulate an operational data ontology, specify data inputs and computational modelling components, and outline validation methods. The focus of the present article is to make the conceptual argument that the existing framework families reviewed here address complementary aspects of QoL but do not, individually, provide the integrated representation identified through the synthesis, and that a paradigm capable of representing continuous, multimodal, integrative and rights-compatible QoL, is needed to enable the field to move from concept to practice.

In addition to making the conceptual argument for a paradigm that represents continuous, multimodal, integrative, and rights-compatible QoL, the conceptual requirements of such a paradigm were derived from this review. These requirements include the ability to integrate data from various sources, i.e., biological, behavioral, social and environmental; the need for continuous and multimodal data collection; the requirement for adaptive personalized support based on individual children’s profiles; the necessity for longitudinal representation of developmental trajectories; the necessity for integrated representations of both child and family QoL; the necessity for foregrounding of child voice and self-determination; and finally, the necessity for rights compliant data governance. A framework meeting these requirements would not replace the many accomplishments documented in this review. Instead, it would build upon those accomplishments and remediate their cumulative shortcomings. To further synthesize the recurring system-level requirements identified across the reviewed literature, Table 1 summarizes the major conceptual, methodological, and implementation gaps that support the need for a more integrated QoL framework.

## 6 Quality of life data use for developmental interventions and policies

### 6.1 Person-centered planning

QoL frameworks represent a fundamental input for person-centered planning (PCP); PCP is a planning approach where supports are developed around an individual’s goals, preferences, and circumstances rather than through pre-established service categories (2, 3). The eight-domain framework provides a useful scaffold to assist planners to address all applicable areas of a child’s life when developing plans, as opposed to focusing only on clinical or academic areas of concern. QoL data can be utilized to identify strength and vulnerability areas in children across domains to support targeted intervention efforts. A child who had high levels of physical well-being and personal development, but lower levels of social inclusion and self-determination, for example, may require intervention efforts related to increasing access to community participation opportunities and enhancing their opportunity to make choices.

### 6.2 Evaluation of interventions

QoL measurements are important indicators for assessing the outcomes of interventions, in conjunction with traditional outcomes measuring symptom reduction or skill acquisition (23, 24). An educational intervention that enhances a child’s academic skills may concurrently decrease QoL if it causes additional stress for the child, restricts opportunities for engaging in preferred activities, or creates negative social interactions. QoL assessments can identify such adverse impacts of interventions and support comprehensive evaluations regarding the overall impact of interventions. For example, McGrath et al. (24) found that the QI-Disability could assess intervention-induced enhancements in QoL for children with complex needs; demonstrating the utility of the QI-Disability in evaluative research contexts.

### 6.3 Program evaluation and continuous quality improvement

At the program level, aggregated QoL data can support continuous quality improvement initiatives and accountability. Service providers can utilize QoL data to identify areas of population strength and weakness, compare performance relative to similar programs, and track trend changes over time (3). Aggregated QoL data showing consistently low scores on social inclusion metrics, for example, may stimulate changes at the program level to increase opportunities for children to participate in community activities. However, utilizing aggregated QoL data to facilitate quality improvement requires aggregation capabilities and integration across the fragmented framework landscape, providing another rationale for developing more integrated frameworks.

### 6.4 Policy accountability and funding

QoL data can also be utilized as evidence to support accountability toward funders, policymakers, and other stakeholders. As value-based care and outcomes-based funding continue to grow in popularity, QoL metrics represent critical information regarding program success that complements traditional process metrics. At the policy level, QoL metrics collected at a population level can identify systemic gaps and inform resource allocations, as well as policy development (3, 7, 29). Comparative analyses across various service delivery models, geographic locations, and policy systems can help identify approaches associated with better QoL outcomes, thus, providing evidence to inform decisions about issues such as inclusive education, respite services or supported decision-making frameworks.

## 7 Future directions and recommendations

### 7.1 Enhancing measurement practices for severely impacted children

Additional methodologic developments are needed to develop QoL measurement practices that meet the needs of children with severe and profound disabilities. Some examples of potential methodologic developments include: behavioral observations focused on engagement and emotional state; multimodal physiological monitoring as objective correlates of states of being; idiographic assessments based on each child’s unique behavioral repertoire; and technology-enhanced methodologies, such as ecological momentary assessment (EMA) and continuous sensing technologies (9–11). Further research should examine how these methodologies might be combined within an overarching framework to support more comprehensive QoL assessment.

### 7.2 Supporting self-reporting and participatory research

When possible, QoL assessments should rely on self-reporting. Various accessible options exist for assisting children with disabilities to report their own QoL, including: using simpler language and response formats; using visual supports; using augmentative and alternative communication (AAC); adapting interview protocols; and creating digital platforms tailored to a child’s neuro-cognitive profile (17). Meaningful involvement of people with disabilities in instrument and framework development is important in order to ensure that assessed dimensions reflect what individuals with disabilities themselves find meaningful; consistent with the “nothing about us without us” principle.

### 7.3 Longitudinal research

Most existing research regarding QoL has been conducted on a cross-sectional basis. Longitudinal research is necessary to understand QoL trajectories throughout childhood and adolescence; determine predictors of positive versus negative trajectories; study relationships between early QoL and later adult outcomes; and provide insight regarding optimal times for implementing interventions (1). Developing frameworks that allow for updating continuously-timestamped data across all relevant categories will greatly contribute to advancing this research agenda.

### 7.4 Cross-cultural research

Additionally, further cross-cultural research is necessary to investigate: how cultural values shape QoL; whether structural aspects of QoL are universally applicable or culturally dependent; how disability experiences differ across cultures; and which policies are most effective in varying contexts (20, 29). While cross-cultural studies typically involve translating instruments and conducting psychometric adaptations, they should ultimately incorporate qualitative and contextual approaches that reflect culturally-grounded perspectives.

### 7.5 Intervention research

While substantial progress has been made in terms of QoL measurement practices, there continues to be a paucity of research regarding interventions intended to improve QoL. Priority areas for intervention research include: examining multi-component interventions that target multiple domains; determining mechanisms underlying interventions; identifying variables that moderate intervention effects; comparative effectiveness studies; and cost-effectiveness analyses (1). Comprehensive frameworks such as those proposed here may support such research by allowing for more detailed assessment and modelling of intervention effects.

## 8 Conclusion

To begin, QoL has evolved into a central organizational construct within both disability research and practice. By offering a holistic perspective centered around the individual child with neurodevelopmental and/or learning disabilities, it provides an opportunity to assess QoL using a person-centered and strength-based framework.

This review examined the evolution of QoL models from deficits-based models to rights-oriented models focusing on strengths. In addition, this review analyzed different framework categories used to measure QoL. Although the most common framework utilized is the Schalock and Verdugo’s eight-domain model, numerous other instruments were identified as being useful for measuring QoL among children with developmental disabilities, including the KidsLife Scale, KidsLife Down Scales, and QI – Disability scale. Additionally, this review discussed the use of technology-supported assessments at both the family-level, and as a means of assessing QoL.

Although there have been significant advancements in QoL research, the comparative analysis identified several recurring limitations across the reviewed frameworks. These included reliance on proxy reports, predominantly cross-sectional or static assessment approaches, fragmentation across populations and conditions, and limited responsiveness to change for some children with significant disabilities. Additional limitations identified across parts of the literature included challenges in cultural adaptability, integration of functional and environmental dimensions, incorporation of rights and self-determination indicators, and integration of contemporary digital technologies into QoL assessment.

Overall, the comparative analysis suggests that no single framework reviewed here fully addresses the range of conceptual, longitudinal, contextual, and rights-related considerations identified across the literature. These findings point to the need for further investigation of more integrated approaches to pediatric QoL assessment. Within this context, the digital twin paradigm is proposed as a candidate conceptual direction because its capacity for continuously updated, individualized, and multimodal representation may correspond to several of the requirements identified through this review. This review therefore provides a conceptual basis for future investigation of such approaches rather than evidence of their effectiveness for pediatric QoL assessment. No study identified in this review applied a digital twin approach to QoL assessment in this population; its implementation and empirical validation therefore remain areas for future research.

The ultimate goal behind the development of such a framework is not to simply create additional measurements for measurement’s sake. Rather it is to establish QoL models that support meaningful and rights-respecting lives for children with neurodevelopmental and learning disabilities and their families.
