# Supplementary material for: Beyond benchmarks: a narrative review of evolving quality of life frameworks for children with neurodevelopmental and learning disabilities and the need for integrated digital twin approaches
Source: Front Public Health. 2026 Sep 30;14:1930098. doi: 10.3389/fpubh.2026.1930098 (PMC13662419; doi:10.3389/fpubh.2026.1930098)
Supplement: Supplementary file 1 [file Data_Sheet_1.PDF]

Beyond Benchmarks: A Narrative Review of Evolving Quality of Life Frameworks for Children with Neurodevelopmental and Learning Disabilities

Search Strategy and Source-Identification Audit

1. Review approach and reporting boundary

This supplementary file documents the database search strategies and source-identification procedures used to support the structured narrative review. The review was designed as a conceptually guided narrative synthesis rather than a systematic or scoping review. Structured search procedures were used to support transparent and reproducible source identification, while the analytical synthesis remained purposive and qualitative.

2. Source-identification audit

The focused database sets used for source identification comprised 473 records from PubMed and 163 records from Scopus. A further 35 records were identified manually through reference and contextual searching. Following consolidation in Zotero, the source-identification dataset comprised 671 records. From this broader candidate pool, 30 articles were purposively retained for the analytical synthesis based on their relevance to the review questions and their conceptual, methodological, or contextual contribution.

Source	Search set / route	Records identified	Role in review	Supporting documentation
PubMed	Set 3 – Targeted conceptual–clinical refinement	473	Focused database set aligned with the review objectives	Search syntax and filters reported below; records summarized in S1
Scopus	Set 2 – Conceptual refinement	163	Focused database set aligned with the conceptual and methodological scope	Search syntax and filters reported below; records summarized in S1
Manual identification	Reference and contextual searching	35	Supplemented database identification with relevant foundational/contextual literature	Records documented in S1
Merged source dataset	Zotero consolidation	671	Consolidated source-identification dataset	Merged dataset documented in S1

Final analytical set	Purposive retention	30	Representative articles retained for conceptual and thematic synthesis	Selected set in S1; inclusion rationale in S2

Note: The counts above describe the documented source-identification pathway. No screening-stage or exclusion counts that were not recorded during the review process have been inferred or retrospectively reconstructed.

3. Database-specific search strategies

3.1 PubMed – Set 3: Targeted conceptual–clinical refinement

Purpose: To identify studies that integrate quality of life as a conceptual construct within clinically grounded pediatric neurodevelopmental and learning-disability contexts, with emphasis on frameworks, models, instruments, and measurement.

Search string: ("Quality of Life"[MeSH] OR "quality of life" OR QoL) AND ("Neurodevelopmental Disorders"[MeSH] OR "learning disabilit*" OR "learning disorder*") AND ("Child"[MeSH] OR "Adolescent"[MeSH]) AND ("Models, Theoretical"[MeSH] OR framework* OR model* OR instrument* OR measurement*)

Focused database-search period: 2015–2025.

Filters: English; Humans; Child (birth–18 years).

Records retrieved: 473.

3.2 Scopus – Set 2: Conceptual refinement

Purpose: To identify conceptual quality-of-life frameworks, models, and measurement instruments relevant to children with neurodevelopmental and learning disabilities.

Search string: ("quality of life" OR QoL OR well-being OR "life satisfaction") AND (neurodevelopmental disorder* OR learning disabilit* OR learning disorder*) AND (child* OR pediatric* OR adolescent*) AND (framework* OR model* OR instrument*)

Focused database-search period: 2015–2025.

Filters: English.

Records retrieved: 163.

Search-period clarification: The 2015–2025 period refers to the focused PubMed and Scopus database searches. The broader narrative synthesis considered literature published during 2007–2026, including relevant foundational literature predating the focused database-search period and subsequently identified literature where necessary to trace the conceptual development of major QoL frameworks and address the review questions.

Broader exploratory searches preceded these focused sets and were used to refine the search strategy. The focused PubMed and Scopus sets reported above are the database sets

represented in the consolidated source-identification dataset documented in Supplementary File S1.

4. Selection and analytical documentation

The focused database records and manually identified sources were consolidated in Zotero to support source selection for the narrative synthesis. Selection was guided by the stated review scope and inclusion/exclusion criteria. The final analytical set comprised 30 articles retained as representative of major conceptual contributions, framework innovations, measurement approaches, and contextual considerations relevant to the review questions.

The analytical workflow was documented through three linked mapping layers: (1) inclusion-rationale mapping, documenting why individual studies were retained; (2) thematic mapping, identifying recurring conceptual and methodological themes; and (3) gap mapping, identifying limitations relevant to future integrative frameworks.

5. Relationship among Supplementary Files S1–S5

01. **S1 – Study Identification and Selection Summary:** documents the database/manual source sets, merged source dataset, and final 30-article analytical set.
02. **S2 – Inclusion Rationale Mapping:** documents the rationale for retaining the selected studies.
03. **S3 – Thematic Mapping Logic:** documents the thematic coding and organization underlying the narrative synthesis.
04. **S4 – Gap Mapping:** documents the mapping of observed limitations to the gap categories used in the synthesis.
05. **S5 – Search Strategy and Source-Identification Audit:** reports the focused database search syntax, filters, retrieval counts, and source-identification pathway.

6. Methodological scope

These structured procedures were used to strengthen transparency and reproducibility of source identification and analytical documentation. The review was not designed as a systematic or scoping review, and no formal study-level quality appraisal or risk-of-bias assessment was undertaken. Accordingly, the findings are interpreted as a conceptual and qualitative synthesis of selected literature rather than as a systematic assessment of the overall strength or certainty of evidence.
